# Commentary: High Expression of Cancer-IgG Is Associated With Poor Prognosis and Radioresistance *via* PI3K/AKT/DNA-PKcs Pathway Regulation in Lung Adenocarcinoma

**DOI:** 10.3389/fonc.2021.741089

**Published:** 2021-10-07

**Authors:** Liu Yang, Pingan Lu, Song Qu

**Affiliations:** ^1^ Department of Radiation Oncology, Guangxi Medical University Cancer Hospital, Nanning, China; ^2^ Amsterdam UMC, Faculty of Medicine, University of Amsterdam, Amsterdam, Netherlands; ^3^ Key Laboratory of High-Incidence Tumor Prevention & Treatment (Guangxi Medical University), Ministry of Education, Nanning, China

**Keywords:** neoplasms, radioresistance, DNA-PK, cancer-derived IgG, normal tissue toxicity

We have read with great interest the article recently published by Yang and colleagues (1), in which surgical tissue specimens from 56 lung adenocarcinoma (LUAD) patients as well as LUAD cell lines H292, A549, H1299 and PC9 were employed to excavate the functional link of cancer-derived IgG with acquired radioresistance. The high expression of cancer-IgG in LUAD tissues and cell lines and its correlation with inferior clinical outcomes (1) concur with previous findings in clear cell renal cell carcinoma (2), ovarian cancer (3), colorectal cancer (4) and salivary adenoid cystic carcinoma (5), which reinforced the concept that cancer-IgG plays an indispensable role in malignant transformation and tumor progression. Furthermore, this study is arguably the first study demonstrating that cancer-IgG overexpression could substantially promote the post-irradiation phosphorylation of DNA-dependent protein kinase catalytic subunit (DNA-PKcs). This valuable finding is in our opinion worth of a further emphasis.

DNA-PK is a key effector of the non-homologous end-joining (NHEJ) pathway, which is the predominant DNA damage repair machinery used by tumor cells to combat ionizing radiation (IR) induced DNA double strand breaks. Over the past decades, myriad research groups and pharmaceutical companies have made great efforts to develop novel pharmaceutical compounds that could potently and specifically inhibit DNA-PK to potentiate IR-induced cytotoxicity in radioresistant neoplasms. However, the cardinal principle of radiation oncology – maximizing dose to tumor while minimizing damage to surrounding normal tissues – has not received sufficient attention in this treatment strategy. As normal cells also need the expression and activation of DNA-PK to survive IR-insult, systemic administration of DNA-PK inhibitors (DNA-PKIs) in combination with radiotherapy may exacerbate normal tissue toxicity as much as tumor toxicity. For example, the development of DNA-PKIs wortmannin and LY294002 has been discontinued at preclinical phase due to severe systemic toxicity in mouse models (6). The same apprehension was further raised by data released from an ongoing phase I trial (NCT02516813) testing the tolerability of DNA-PKI M381 in combination with radiotherapy, which reported several forms of normal tissue toxicity including dysphagia, prolonged mucosal inflammation/stomatitis and radiation skin injury (7). Unsurprisingly, these normal tissue reactions were not observed in another phase I trial (NCT02316197) evaluating the tolerability of M381 as monotherapy (8).

Currently, attempts towards finding novel treatment strategies that selectively target the NHEJ-mediated DSB repair in tumors are still ongoing, exemplified by the DNA-PKI prodrug that could be selectively activated in the hypoxic tumor microenvironment (9). In this regard, we would like to voice the great significance of the findings by Yang and colleagues (1). Since cancer-IgG is specifically secreted by cancer cells and is capable of promoting the phosphorylation of DNA-PKcs, targeted pharmacological inhibition of cancer-IgG holds the promise to significantly circumvent the problematic normal tissue toxicities of traditional DNA-PKIs while providing similar radiosensitizing effects. Additionally, as cancer-IgG expression was not only observed in LUAD (2–5), multifaceted therapeutic benefits of anti-cancer-IgG agents could also be expected for radioresistant patients with other malignant diseases, as well as for patients suffering from intrinsic or acquired resistance to DNA-damaging chemotherapeutics.

## Author Contributions

LY and PL performed literature research and drafted the manuscript. SQ supervised the study and reviewed the manuscript. All authors contributed to the article and approved the submitted version. LY and PL contributed equally as co-first authors.

## Conflict of Interest

The authors declare that the research was conducted in the absence of any commercial or financial relationships that could be construed as a potential conflict of interest.

## Publisher’s Note

All claims expressed in this article are solely those of the authors and do not necessarily represent those of their affiliated organizations, or those of the publisher, the editors and the reviewers. Any product that may be evaluated in this article, or claim that may be made by its manufacturer, is not guaranteed or endorsed by the publisher.
